# Safety of inactivated SARS-CoV-2 vaccines in myasthenia gravis: A survey-based study

**DOI:** 10.3389/fimmu.2022.923017

**Published:** 2022-08-05

**Authors:** Hong-Yan Li, Li-Yuan Shao, Min Song, Shi-Min Hu, Yao-Xian Yue, Hai-Feng Li

**Affiliations:** ^1^ Department of Neurology, Qilu Hospital (Qingdao), Cheeloo College of Medicine, Shandong University, Qingdao, China; ^2^ Department of Neurology, Xuanwu Hospital, Capital Medical University, Beijing, China

**Keywords:** safety, myasthenia gravis, SARS-CoV-2, vaccination, survey

## Abstract

**Background:**

Vaccination remains the most effective measure to prevent SARS-CoV-2 infection and worse outcomes. However, many myasthenia gravis (MG) patients are hesitant to receive vaccine due to fear of worsening.

**Methods:**

MG patients were consecutively enrolled in two MG centers in North China. The “worsening” after vaccination was self-reported by MG patients, and severity was measured with a single simple question. The general characteristics and disease status immediately prior to the first dose were compared between the worsening and non-worsening groups. Independent factors associated with worsening were explored with multivariate regression analysis.

**Results:**

One hundred and seven patients were included. Eleven patients (10.3%) reported worsening after vaccination, including eight patients with mild or moderate worsening and three patients with severe worsening. Only one of them (0.9%) needed an escalation of immunosuppressive treatments. There were significant differences between the worsening and non-worsening groups in terms of Myasthenia Gravis Foundation of America classes immediately before the first dose and intervals since the last aggravation. Precipitating factors might contribute to the worsening in some patients. Logistic regression revealed that only interval since the last aggravation ≤6 months was associated with worsening after SARS-CoV-2 vaccination (*P* = 0.01, OR = 8.62, 95% CI: 1.93–38.46).

**Conclusion:**

SARS-CoV-2 vaccines (an overwhelming majority were inactivated vaccines) were found safe in milder Chinese MG patients who finished two doses. Worsening after vaccination was more frequently seen in patients who were presumed as potentially unstable (intervals since last aggravation ≤6 months). However, mild worsening did occur in patients who were presumed to be stable. Precipitating factors should still be sought and treated for better outcome.

## Introduction

The coronavirus disease 2019 (COVID-19) is still raging worldwide. Vaccination remains the most effective measure to prevent a SARS-CoV-2 infection and its worse outcomes (1). Several studies have reported the safety of SARS-CoV-2 vaccines in myasthenia gravis (MG) patients (2–5). However, many MG patients are hesitant to receive the vaccine for fear of worsening (6). It is important to know the clinical characteristics of patients who are prone to worsening in order to provide individualized advice. A retrospective analysis was conducted in MG patients who received SARS-CoV-2 vaccines from two MG centers in China to report the safety of SARS-CoV-2 vaccination and to explore the factors which might indicate possible worsening.

## Materials and methods

This study was conducted from March 2021 to March 2022 in Qilu Hospital (Qingdao) and Xuanwu Hospital to investigate the possible worsening of MG after SARS-CoV-2 vaccination. The eligible patients should be diagnosed as MG at least 1 month before being included. We designed and sent a structural questionnaire (**Supplementary Material 1**) to the patients in our registry and confirmed their replies by WeChat (an instant chat tool) or telephone or by a face-to-face visit if needed. We also used this questionnaire in the follow-up of patients who reported worsening.

The “worsening” after vaccination was self-reported by MG patients, which was defined as the occurrence of additional symptoms or the aggravation of existing symptoms in at least one of the six muscle groups (extraocular, facial, bulbar, cervical, limb and respiratory muscles) within 28 days after each vaccination dose. When patients reported worsening, they were required to recall the symptoms and overall severity immediately prior to the first dose as the baseline severity. The overall severity at baseline and maximal worsening after vaccination was determined with a single simple question (what percentage of normal do you feel regarding your severity of MG symptoms, 0–100% of normal), which had been shown to correlate strongly with limb muscle weakness and moderately with bulbar and respiratory symptoms (7). The “normal” refers to a patient’s sense of well-being (100%); the greater is the %, the mild is the worsening. The patients were being kept in close communication to determine whether worsening was related to MG and to find and manage any significant impact on their daily life until they recovered to the status prior to vaccination. If the patients reported a significant impact on their daily life within 14 days after worsening, a face-to-face visit was arranged by the treating neurologists for exclusion of the effects of concurrent diseases and for the determination of escalation in immunosuppressive treatment (IST). The final information of the survey was recorded in our database after confirmation of the self-reports of the patients.

Data on general characteristics such as gender, onset age, thymoma, acetylcholine receptor (AChR) antibody or muscle-specific kinase (MuSK) antibody, muscle involvement (ocular/generalized) at onset, and maximal Myasthenia Gravis Foundation of America (MGFA) classes during total MG duration until the first dose were extracted from our MG database. The status immediately before the first dose was acquired from the MG database and by active communication with the patients, including the age when they received the first dose, MGFA classes immediately before the first dose (IBFD MGFA class), post-intervention status immediately before the first dose (IBFD PIS) (8), Myasthenia Gravis Status and Treatment Intensity (MGSTI) levels (9), and the interval since last aggravation to the first dose. The IBFD PIS was defined by only impacts on daily life of the patients without considering the persistence of this status. MGSTI contained six levels: level 0 refers to complete stable remission (CSR), level 1–3 refers to pharmacologic remission (PR) or minimal manifestation (MM) with mono or dual oral therapies of glucocorticoids and immunosuppressants, and level 4 refers to symptomatic but not requiring IV immunoglobulin, plasma exchange, or hospitalization. The PIS used in MGSTI levels was consistent with the definition of PIS by MGFA (8), which required at least 1 year for being classified as CSR, PR, and MM. We used the cutoff of the MGSTI score in reference to the study of rituximab in anti-MuSK MG patients (9). Last aggravation referred to the nearest worsening which needs escalation of ISTs in patients with a total duration >6 months or the onset of MG in patients with a total duration ≤6 months. By this definition, the status of the patients during the intervals is improved or unchanged or even worsened slightly but need no escalation of ISTs. Precipitating factors (infection, fatigue, emotional stress, suspected drug use, *etc.*), IST escalation, and intervals between the worsening after vaccination and recovery to the status prior to vaccination were recorded. This study was approved by the Ethics committee of both hospitals, and informed consents were obtained from the patients or their authorized family members.

### Statistical analysis

Continuous variables were expressed as mean ± standard deviation (SD) or median (interquartile range) and compared using *t*-test or the Mann–Whitney *U*-test. Categorical variables were expressed as frequencies (percentages) and compared using the chi-square test or Fisher’s exact test. Logistic regression was conducted to determine the independent factors associated with worsening. Statistical analyses were performed using IBM SPSS, version 20.0 (SPSS Inc., Chicago, IL, USA). A two-tailed *p <*0.05 was considered significant.

## Results

A total of 107 MG patients received at least one dose of SARS-CoV-2 vaccine, 105 patients received homologous inactivated vaccines, and two patients received homologous recombinant subunit vaccines. Ninety-seven patients received the second dose at 26 (22–32) days after the first dose, and 35 patients received the third dose at 187 (184–200) days after the second dose. The first dose was given to 22 patients with interval since last aggravation ≤6 months (20.6%), including 10 patients whose total MG duration was ≤6 months. Their IBFD PIS at first dose was MM or better in 10 patients and inferior to MM in 12 patients. There were no SARS-CoV-2 infections in all patients during the follow-up. The detailed baseline information and the status immediately before the first dose are shown in **Table 1**.

**Table 1:** Comparison between non-worsening and worsening patients.

	Total (*n* = 107)	Non-worsening (*n* = 96)	Worsening (*n* = 11)	*P*-value
General characteristics				
Gender				
Male	54/107 (50.5%)	48/96 (50%)	6/11 (54.5%)	0.78
Female	53/107 (49.5%)	48/96 (50%)	5/11 (45.5%)	
Onset age	38.79 ± 1.73 (range: 2–73)	37.75 ± 18.01 (range: 2–68)	47.91 ± 14.35 (range: 18–73)	0.09
Onset involvement				
Ocular	73/107 (68.2%)	64/96 (66.7%)	9/11 (81.8%)	0.26
Generalized	34/107 (31.8%)	32/96 (33.3%)	2/11 (18.2%)	
Thymoma				
Positive	17/107 (15.9%)	16/96 (16.7%)	1/11 (9.1%)	0.45
Negative	90/107 (84.1%)	80/96 (83.3%)	10/11 (90.9%)	
Antibodies				
AChR (+)	67/104 (64.4%)^a^	60/93 (64.5%)	7/11 (63.6%)	0.6
MuSK (+)	4/104 (3.8%)^b^	4/93 (4.3%)	0/11 (0)	0.64
Maximal MGFA class within total duration				
I	30/107 (32.6%)	38/96 (39.6%)	3/11 (27.3%)	0.65
II	35/107 (38%)	32/96 (33.3%)	4/11 (36.4%)	
III	9/107 (9.8%)	10/96 (10.4%)	2/11 (18.2%)	
IV	11/107 (12%)	9/96 (9.4%)	2/11 (18.2%)	
V	7/107 (6.5%)	7/96 (7.3%)^c^	0/11 (0)	
Status immediately before first dose				
Age	45.68 ± 1.49 (range: 12–75)	44.52 ± 15.16 (range: 12–71)	55.82 ± 14.72 (range: 32–75)	0.87
IBFD MGFA class				
Asymptomatic	41/107 (38.3%)	39/96 (40.6%)	2/11 (18.2%)	0.01^d^
I	29/107 (27.1%)	25/96 (26%)	4/11 (36.4%)	
IIa	30/107 (28%)	28/96 (29.2%)	2/11 (18.2%)	
IIb	6/107 (5.6%)	4/96 (4.2%)	2/11 (18.2%)	
IIIa	1/107 (0.9%)	0/96 (0)	1/11 (9.1%)	
IBFD PIS				
MM or better	82/107 (76.6%)	76/96 (79.2%)	6/11 (45.5%)	0.08
Inferior to MM	25/107 (23.4%)	20/96 (20.8%)	5/11 (54.5%)	
MGSTI score				
Levels 0–2	68/99 (68.7%)	62/88 (70.5%)	6/11 (45.5%)	0.31
Levels 3-4	31/99 (31.3%)	26/88 (29.5%)	5/11 (54.5%)	
Interval since last aggravation				
>6 months	85/107 (79.4%)	79/96 (82.3%)	6/11 (54.5%)	0.046^e^
≤6 months	22/107 (20.6%)	17/96 (17.7%)	5/11 (45.5%)	
Total duration to first dose	64 (25–121) (range: 1–389)	57.5 (25–118.75) (range: 1–389)	108 (31–157) (range: 6–180)	0.38

IBFD, immediately before the first dose.

a Three patients had not tested antibodies.

b There were no patients with both AChR and MuSK antibodies.

c Need ventilation previously; all occurred 49–211 months before the first dose. IBFD PIS was MM or better in four patients. IBFD MGFA classes were asymptomatic in one patient and IIa in six patients.

d Significantly different between non-worsening and worsening group (*P* = 0.01).

e Significantly higher incidence of worsening in patients whose interval since last aggravation ≤6 months (*P* = 0.046, OR = 3.87, 95% CI: 1.06–14.17).

Eleven patients (10.3%) reported 12 episodes of worsening after COVID-19 vaccination, including six (5.6%) after the first dose and six (6.2%) after the second dose. No patient reported worsening after the third dose. Since the first and the second doses were in close time interval and most of the patients finished two doses, the baseline data of patients who received the first dose were used in the subsequent analysis. Six patients were examined face to face by the treating neurologists when their worsening lasted more than 14 days or when they visit for routine follow-up, and the other five patients who recovered before 14 days were evaluated based on the online survey. There was a significant difference in IBFD MGFA classes between the worsening and non-worsening groups (*P* = 0.01). A significantly higher incidence of worsening was noted in patients with interval since last aggravation ≤6 months (*P* = 0.046, OR = 3.87, 95% CI: 1.06–14.17). Moreover, there were marginal differences in onset age and IBFD PIS (MM or better) between the two groups. Logistic regression was conducted by taking worsening as dependent variable and patient characteristics (gender, onset age, onset muscle involvement, thymoma, and AChR or MuSK antibodies), age at first dose, interval since last aggravation, IBFD MGFA classes, IBFD PIS, and MGSTI score as independent variables, and it was revealed that interval since last aggravation ≤6 months was the only independent factor associated with worsening after vaccination (*P* = 0.01, OR = 8.62, 95% CI: 1.93–38.46).

Details on the 11 patients who reported worsening are presented in **Table 2**. Eight patients (including one who suffered two worsening episodes after each dose) suffered from mild worsening (50–80% of normal), two of whom reported precipitating factors. Four patients recovered to the status prior to vaccination without IST escalation within 14 days. Two patients reported moderate worsening severity at 40 or 20% of normal, and they had pneumonia at 3 days after the first dose or an upper respiratory infection at 5 days before the first dose. Worsening occurred 18 days or 1 day after vaccination. The interval since last aggravation was 31 months in one patient and 49 months in the other, and MGSTI was level 0 in one patient and level 1 in the other. Treated with antibiotics, they recovered in 7 and 14 days, respectively. Only one patient needed an escalation of prednisone dosage and an addition of tacrolimus 10 days after worsening, which was determined by the treating neurologist. She had suffered from long-term exhaustion before the first dose and reported 20% of normal. The interval since last aggravation was 41 months, and the MGSTI was level 2. She recovered 2 months later (**Table 2**).

**Table 2:** Details on the 11 patients with worsening.

Patient^a^	AChRAb^b^	Baseline factors prior to the first dose						Worsening and prognosis					
		Total duration^c^	Treatment^d^	IBFD MGFA class	IBFD PIS	MGSTI score	Interval ≤6 months	Worsening symptoms	Severity^e^	Vaccine dose	Precipitating factors	Treatment	Intervals to recovery^f^
No. 1 52, F	+	115	Pre, 35 mg/day	IIIa	Inferior to MM	Level 4	+	Diplopia	80%	2nd	None	IST^g^ unchanged	2 weeks
No. 2 69, F	+	166	Pre, 2.5 mg/day; CsA, 50 mg/day	IIa	Inferior to MM	Level 4	–	Ptosis, diplopia	70%	1st	None	IST unchanged	1 month
No. 3 50, M	–	86	Pre, 20 mg/day	I	Inferior to MM	Level 4	+	Difficulty in arm raising	70%	2nd	None	IST unchanged	1 week
No. 4 75, F	+	168	Pre, 5 mg/day	I	MM1	Level 1	–	Ptosis, diplopia	70%	2nd	Fatigue, emotional stress	IST unchanged	1 month
No. 5 48, M	+	113	None presently	Asymptomatic	CSR	Level 0	–	Ptosis, diplopia	70%	1st and 2nd	Fatigue	None	3 and 7 days
No. 6 49, M	–	32	Not ever treated with IST	I	MM0	Level 0	–	Ptosis	70%	1st	None	None	1 week
No. 7 42, M	–	14	PB, 180 mg/day	IIb	Inferior to MM	Level 4	+	Difficulty in head raising and swallowing	50%	2nd	None	IST unchanged	1 week
No. 8 49, M	+	72	Pre, 50 mg/day	IIa	Inferior to MM	Level 4	+	Ptosis, diplopia	50%	1st	None	IST unchanged	2 weeks
No. 9 75, F	+	40	Not ever treated with IST	IIb	MM0	Level 0	+	Dysphagia, ptosis	40%	1st	Pneumonia	Antibiotics only	1 week
No. 10 73, M	+	182	Pre, 2.5 mg/day	I	MM1	Level 1	–	Nasal speech, dyspnea	20%	1st	Upper respiratory infection	IST unchanged, antibiotics	2 weeks
No. 11 32, F	–	178	Pre, 15 mg/day	Asymptomatic	MM1	Level 2	–	Ptosis, diplopia	20%	2nd	Fatigue	IST escalation	2 months

IBFD, immediately before the first dose; Pre, prednisone; PB, pyridostigmine bromide; CsA, cyclosporin A; ISTs, immunosuppressive treatments.

a The age when receiving the first dose.

b AChR antibody; no patient had a positive MuSK antibody.

c Disease duration from disease onset to receiving the first dose (months).

d Treatment immediately prior to vaccination.

e Reflected by “what percentage of normal do you feel regarding your maximal severity of worsening”, 0–100% of normal.

f The interval between worsening and recovery to prior status before vaccination.

g ISTs referred to immunosuppressive treatment, including prednisone and non-steroidal immune therapies.

## Discussions

The safety profile of MG patients after SARS-CoV-2 vaccination could be partly learned from the experiences of SARS-CoV-2 vaccination in autoimmune diseases, such as rheumatic disease, as well as experiences of influenza vaccines in MG patients. In 2021, an international registry including 5,121 inflammatory/autoimmune rheumatic and musculoskeletal disease (I-RMDs) patients who received SARS-CoV-2 vaccination reported that an overwhelming majority of the patients tolerated the vaccination well, with rare reports of worsening (4.4%, including 0.6% severe and 1.5% requiring medication changes) (10). In MG patients, no exacerbation of MG was found after influenza vaccination in most studies (11–14). However, aggravation which needed IST escalation did occur as a rare event (2/133, 1.5%) (15).

There have been several reports on the worsening of MG following SARS-CoV-2 vaccines. One case report described a myasthenic crisis after SARS-CoV-2 vaccination (16). In 294 MG patients who received mRNA vaccines in Japan (3), three patients (1%) were hospitalized for IVIg and/or methylprednisolone pulse; however, the number of patients with mild worsening was not reported. In 55 MG patients who received mRNA vaccines in Israel (4), eight patients (14.5%) reported worsening, and only three patients (5.5%) required additional medication to treat their symptoms. In 104 MG patients who received mRNA vaccines in Italy (5), eight patients (7.7%) reported worsening, including two patients (1.9%) who needed IST escalation. Chang et al. (2) reported 22 Chinese MG patients who received SARS-CoV-2 vaccines (21 inactivated vaccines and one recombinant subunit vaccines)—18 patients had complete resolution of symptoms for at least 2 months, three were in MGFA I class, and one was in MGFA II class before vaccination; only two patients (9.1%) reported mild worsening which resolved quickly within a few days without IST escalation. In our study, no patient received mRNA SARS-CoV-2 vaccines. In our study, 11 patients (10.3%) reported worsening, and only one patient (0.9%) was confirmed as aggravation which needed IST escalation. Our results were consistent with the abovementioned results in the overall safety profiles of vaccination in MG, although different vaccines were given.

In this study, with the structured questionnaire, MG worsening was identified by additional symptoms or changes of severity in previous symptoms, which were ascertained by close communication or face-to-face visit. The single simple question provided a simple, quick, and valid evaluation of the worsening severity. Therefore, our survey was feasible on the background of SARS-CoV-2 pandemic. Our database provided detailed information to analyze the factors that might be associated with worsening after vaccination. Status immediately prior to vaccination is most important. The interval since last aggravation (aggravation or onset) ≤6 months prior to vaccination is presumed as possible instability. During this time, the patients might hesitate to receive vaccination. The IBFD MGFA class reflects the severity on vaccination, and the IBFD PIS reflects the impacts of MG on daily life prior to vaccination. The MGSTI score reflects the stability of MG and the ongoing treatment potency. Our result showed that the severity and impact of MG on daily life immediately prior to vaccination and the potential instability status were associated with worsening after vaccination. Logistic regression analysis revealed that the only independent factor for worsening following vaccination was a potentially unstable status. There was no association between worsening and other factors, such as gender, onset age, pathogenic antibodies, thymoma, onset muscle involvement, and maximal MGFA classes. Even seven patients who previously needed invasive ventilation 49–211 months before the first dose reported no worsening after vaccination. Moreover, the comprehensive status of stability and treatment potency (MGSTI levels) were not different between the worsening and non-worsening groups. This indicated that the potential short-term instability of MG instead of ongoing stability of MG was associated with worsening after vaccination.

There were some limitations in this study. First, this was a retrospectively real-world study, which rendered inevitable selection bias by including most mild patients due to the reluctance of the treating physician to give vaccination in more severe patients. Second, the number of patients who had received three doses was small. Lumping 10 patients who only received one dose and 97 patients who received two doses into one cohort might underestimate the incidence of worsening. However, the present result could indicate the incidence of worsening after two doses and the factors associated with worsening in light of their effect sizes. Third, we lack the data of precipitating factors in 96 non-worsening patients due to difficulty in acquiring these data through this online survey. The precipitating factors were acquired after asking about every detail of the 11 worsening patients.

## Conclusion

SARS-CoV-2 vaccines (an overwhelming majority were inactivated vaccines) were found safe in milder Chinese MG patients who finished two doses. Worsening was more frequently seen in patients who were presumed as potential unstable (duration from last aggravation or onset ≤6 months). However, even patients who were presumed as stable could have mild worsening after vaccination. Precipitating factors should still be sought and treated if needed.

## Data availability statement

The original contributions presented in the study are included in the article/**Supplementary Material**. Further inquiries can be directed to the corresponding authors.

## Ethics statement

The studies involving human participants were reviewed and approved by the ethics committee of Qilu Hospital (Qingdao), Cheeloo College of Medicine, Shandong University and Ethics Committee of Xuanwu Hospital, Capital Medical University. Written informed consent to participate in this study was provided by the participants’ legal guardian/next of kin. Written informed consent was obtained from the individual(s) and minor(s)’ legal guardian/next of kin for the publication of any potentially identifiable images or data included in this article.

## Author contributions

Y-XY and H-FL conceptualized and designed the study and revised the manuscript. H-YL and L-YS interpreted the data and wrote the manuscript. H-YL and S-MH performed statistical analysis. L-YS and MS maintained the research database. All authors contributed to the article and approved the submitted version.

## Funding

This study was supported by the National Natural Science Foundation of China (nos. 81771362 and 82171397 to H-FL) and Research Grant from Qilu Hospital (Qingdao), Cheeloo College of Medicine, Shandong University (QDKY2021RX06 to Y-XY).

## Conflict of interest

The authors declare that the research was conducted in the absence of any commercial or financial relationships that could be construed as a potential conflict of interest.

## Publisher’s note

All claims expressed in this article are solely those of the authors and do not necessarily represent those of their affiliated organizations, or those of the publisher, the editors and the reviewers. Any product that may be evaluated in this article, or claim that may be made by its manufacturer, is not guaranteed or endorsed by the publisher.
