# Supplementary material for: Safety of inactivated SARS-CoV-2 vaccines in myasthenia gravis: A survey-based study
Source: Front Immunol. 2022 Aug 5;13:923017. doi: 10.3389/fimmu.2022.923017 (PMC9388926; doi:10.3389/fimmu.2022.923017)
Supplement: Supplementary file 1 [file DataSheet_1.docx]

Age at 1^st^ dose:

Gender: 🞎 Male 🞎 Female

Date of 1^st^ dose: MM/DD/YY

Type of the vaccine in 1^st^ dose:

🞎 Inactivated vaccine 🞎 Recombinant subunit vaccine 🞎 mRNA vaccine

Adverse reaction within 1 week after 1^st^ dose:

🞎 None

🞎 Redness, induration, pain at the injection site

🞎 Fever

🞎 Fatigue, weakness

🞎 Nausea, loss of appetite

🞎 Joint pain

🞎 Headache

🞎 Others

**Symptoms of MG of maximal severity within 4 weeks after 1^st^ dose**

(Please select ***the classes*** and the ***detailed items*** listed under the classes. If you found additional symptoms or aggravation of existing symptoms, please specify the symptoms immediately prior to the vaccination in a separate sheet below):

🞎 None

🞎 Ocular muscles: 1. ptosis; 2. diplopia; 3. strabismus

🞎 Facial muscles: 1. weakness in eyelid closure; 2. difficulty in blowing cheeks

🞎 Bulbar muscles: 1. difficulty in chewing; 2. slurred or nasal speech; 3. difficulty in expectoration; 4. nose regurgitation; 5. difficulty in swallowing; 6. choking and/or coughing after drinking

🞎 Neck muscles: 1. neck ache when tired; 2. difficulty in head-raising

🞎 Limb muscles: 1. difficulty in arm-raising; 2. difficulty in gripping; 3. finger clumsy or weakness; 4. difficulty in going upstairs; 5. difficulty in standing up from chairs

🞎 Respiratory muscle: 1. short of breath after activity; 2. breathlessness

🞎 Others

**What percentage of normal do you feel regarding your maximal severity of worsening after vaccination?**

|  | |  | |  | |  | |  | |  | |  | |  | |  | |  | |
| --- | --- | --- | --- | --- | --- | --- | --- | --- | --- | --- | --- | --- | --- | --- | --- | --- | --- | --- | --- |
|  | 10 | | 20 | | 30 | | 40 | | 50 | | 60 | | 70 | | 80 | | 90 | |  |

**Immunosuppressive treatments immediately prior to the vaccination**

| 🞎 Prednisone (or equivalent) | dosage__________ |
| --- | --- |
| 🞎 Azathioprine | dosage__________ |
| 🞎 Methotrexate | dosage__________ |
| 🞎 Tacrolimus | dosage__________ |
| 🞎 Ciclosporin | dosage__________ |
| 🞎 Cyclophosphamide | dosage__________ |
| 🞎 Mycophenolate Mofetil | dosage__________ |
| 🞎 IV immunoglobulin (IVIg) |  |
| 🞎 Plasma exchange (PLEX) |  |

**Previous symptoms immediately prior to the vaccination:**

🞎 None

🞎 Ocular muscles: 1. ptosis; 2. diplopia; 3. strabismus

🞎 Facial muscles: 1. weakness in eyelid closure; 2. difficulty in blowing cheeks

🞎 Bulbar muscles: 1. difficulty in chewing; 2. slurred or nasal speech; 3. difficulty in expectoration; 4. nose regurgitation; 5. difficulty in swallowing; 6. choking and/or coughing after drinking

🞎 Neck muscles: 1. neck ache when tired; 2. difficulty in head-raising

🞎 Limb muscles: 1. difficulty in arm-raising; 2. difficulty in gripping; 3. finger clumsy or weakness; 4. difficulty in going upstairs; 5. difficulty in standing up from chairs

🞎 Respiratory muscle: 1. short of breath after activity; 2. breathlessness

🞎 Others

**What percentage of normal do you feel regarding your severity immediately prior to the vaccination?**

|  | |  | |  | |  | |  | |  | |  | |  | |  | |  | |
| --- | --- | --- | --- | --- | --- | --- | --- | --- | --- | --- | --- | --- | --- | --- | --- | --- | --- | --- | --- |
|  | 10 | | 20 | | 30 | | 40 | | 50 | | 60 | | 70 | | 80 | | 90 | |  |

**Possible precipitating factors (only for patients with worsening after vaccination):**

🞎 Infection: 1. upper respiratory tract; 2. gastrointestinal tract; 3. urinary tract infection; 4. others__________

🞎 Fatigue

🞎 Emotional stress

🞎 Possible drugs______________

🞎 Others ______________

*The survey on worsening after the 2^nd^ and 3^rd^ doses are same as the 1^st^ dose, and omitted here.*
